# Managing Polyploidy in *Ex Situ* Conservation Genetics: The Case of the Critically Endangered Adriatic Sturgeon (*Acipenser naccarii*)

**DOI:** 10.1371/journal.pone.0018249

**Published:** 2011-03-29

**Authors:** Leonardo Congiu, Jose Martin Pujolar, Anna Forlani, Silvia Cenadelli, Isabelle Dupanloup, Federica Barbisan, Andrea Galli, Francesco Fontana

**Affiliations:** 1 Dipartimento di Biologia, Università di Padova, Padova, Italy; 2 Istituto Sperimentale Lazzaro Spallanzani, Rivolta d'Adda, Italy; 3 Institute of Ecology and Evolution, University of Bern, Bern, Switzerland; 4 Dipartimento di Biologia, Università di Ferrara, Ferrara, Italy; Montreal Botanical Garden, Canada

## Abstract

While the current expansion of conservation genetics enables to address more efficiently the management of threatened species, alternative methods for genetic relatedness data analysis in polyploid species are necessary. Within this framework, we present a standardized and simple protocol specifically designed for polyploid species that can facilitate management of genetic diversity, as exemplified by the ex situ conservation program for the tetraploid Adriatic sturgeon *Acipenser naccarii*. A critically endangered endemic species of the Adriatic Sea tributaries, its persistence is strictly linked to the *ex situ* conservation of a single captive broodstock currently decimated to about 25 individuals, which represents the last remaining population of Adriatic sturgeon of certain wild origin. The genetic variability of three F1 broodstocks available as future breeders was estimated based on mitochondrial and microsatellite information and compared with the variability of the parental generation. Genetic data showed that the F1 stocks have only retained part of the genetic variation present in the original stock due to the few parent pairs used as founders. This prompts for the urgent improvement of the current F1 stocks by incorporating new founders that better represent the genetic diversity available. Following parental allocation based on band sharing values, we set up a user-friendly tool for selection of candidate breeders according to relatedness between all possible parent-pairs that secures the use of non-related individuals. The approach developed here could also be applied to other endangered tetraploid sturgeon species overexploited for caviar production, particularly in regions lacking proper infrastructure and/or expertise.

## Introduction

Management of captive breeding programs require routinely use of parentage analysis and, to a lesser extent, estimators of relatedness in order to evaluate the relationship among founders in the absence of parentage data. Pedigree information on all aquaculture broodstock is necessary for the estimation and management of inbreeding. When aiming at either livestock breeding or wildlife conservation, it is essential to control and minimize the rate of inbreeding, and therefore avoid mating among close relatives. Parentage assignment using molecular markers implies that parents and offspring are all genotyped at a given number of unlinked genetic markers, and that the information on genotypes is used to assign progeny to the correct parental pair [1]. Pedigree-based genetic management practices have mostly focused on diploid organisms where interpretation and analysis of genetic data is relatively easy due to the simple Mendelian inheritance [2]. Relatedness estimation methods for polyploid species are less straightforward because of the non-Mendelian patterns of segregation. Thus, alternative methods are necessary for genetic relatedness analysis in conservation programs for polyploid species.

This holds particularly true for sturgeons (order Acipenseriformes), of which about 50% of the species are polyploid. Sturgeons were once widely distributed and abundant in the Holarctic region but today exist in the wild only as fragmented and isolated populations with a limited geographic distribution [3], [4]. In the recent press release of 18 March 2010, IUCN identified sturgeons as the most endangered group of species, with 85% of sturgeons being at risk of extinction according to the Red List of Threatened Species (http://www.iucnredlist.org). Since 1998, international trade in all species of sturgeons has been regulated under CITES (http://www.cites.org) owing to concerns over the impact of unsustainable harvesting of and illegal trade in sturgeon populations in the wild. Despite protective fishing regulations, sturgeon populations have been decimated due to human activities including historic exploitation for caviar (unfertilized sturgeon eggs), poaching, damming of rivers and habitat degradation [5]. The dramatic decline of natural sturgeon populations in recent years prompted conservation efforts for most sturgeon species by means of restocking practices. However, procuring an adequate number of breeders might be difficult due to the scarcity of animals in the wild. Thus, the establishment of captive broodstocks for *ex situ* conservation is strongly encouraged through the reproduction of the few wild breeders available and the subsequent rearing of the progenies [6]. However, some particular life-history traits of sturgeons that have proven adaptive over the last 100 million years are now a disadvantage in the face of recent anthropogenic pressures: sturgeons are long-lived organisms that can live up to 100 years in some species, show late maturation (5 to more than 30 years) and do not reproduce annually (2 to more than 10 years between spawning cycles). Moreover, the tetraploid nature of many sturgeon species and the still unknown pattern of chromosomal segregation (double disomic versus tetrasomic) complicates the analytical approach [7].

Our species of interest is the Adriatic sturgeon (*Acipenser naccarii*) a tetraploid species endemic for the North Adriatic region [8]. Once widely distributed in nearly all tributaries of the North Adriatic Sea, the Adriatic sturgeon is currently considered to be at risk of extinction. The species is included in Appendix II of the Convention on international Trade of Endangered Species (CITES; http://www.cites.org) and its status was updated in 2010 from “Vulnerable” to “Critically Endangered” by the IUCN (http://www.iucnredlist.org). In common with most Eurasian sturgeons, catch data show a dramatic population decline from over 2,000 kg per year in the early 1970s to about 200 kg per year in the early 1990s to only 19 individuals caught in 1993 [9].

A breeding program for the Adriatic sturgeon was initiated in 1977 with the transfer of immature wild specimens from the Po River (Northern Italy) to the fish plant Azienda Agricola VIP (Orzinuovi, Brescia, Italy). Following the successful reproduction in captivity of the Adriatic sturgeon for the first time in 1988, restocking practices were conducted in the period 1988–2009 in tributaries of the Po river [10], [11]. Despite releasing about 250, 000 juveniles, recaptures have been scarce and up to now there is no evidence of natural reproduction in the Po river and its tributaries [12]. At present, the remaining F0 parents from the Azienda Agricola VIP stock constitute the only living Adriatic sturgeons of unequivocal wild origin left. All the Adriatic sturgeons reared in Europe for aquaculture purposes directly descend from this limited stock. No data are available from former spawning areas outside Italy, including the Buna river population in Albania, where the presence of the species was confirmed in the late 1990s [13]. Given the scarcity of F0 individuals, *ex situ* conservation in the future depends on the establishment of F1 broodstocks. Considering this, several broodstocks obtained by artificial reproduction at Azienda Agricola VIP have now reached sexual maturity. Three of those F1 groups identified as potential breeders are now reared at three different sites, Treviso, Piacenza and Orzinuovi (at the same Azienda Agricola VIP), funded by local administrations, and are composed by about 100–200 animals each. Unfortunately, until now the breeding program has been conducted without any genetic input and breeders used for artificial reproduction were randomly paired. No information is available on how many or which F0 parent pairs were used to produce these F1 animals and, consequently, relatedness between individuals is unknown.

In the only available study on the evolutionary history of the Adriatic sturgeon, Ludwig *et al*. [12] examined a subset of the parental F0 Azienda Agricola VIP stock (N =  31) together with a sample from the Buna river (Albania, N =  17). On the basis of 987 bp of the mitochondrial DNA (mtDNA) control region, a highly significant genetic differentiation was found between Po and Buna rivers. Within the Po River, mtDNA variation revealed two distinguished haplogroups (Po1 and Po2). Interestingly, Po2 haplotypes were more closely related to the sister species *A. gueldenstaedtii* haplotypes, pointing to a possible ancient introgression episode. However, nuclear variation using microsatellite and AFLP (Amplified Fragment Length Polymorphism) analysis did not support the Po group subdivision while confirming the differentiation between Po and Buna rivers.

The present study characterized the distribution of relatedness values within all remaining Adriatic sturgeons of certain wild origin. This included most of the animals that were employed by Azienda Agricola VIP as F0 parents back in 1977, even if presently dead, and whose progeny was either released in the restocking practices conducted in the period 1988–2009 or reared to be part of the F1 stocks currently available as future source of breeders. Ultimately, we propose a standardized method for the analysis of genetic relatedness data in polyploid species to be used as a simple approach to support practical breeding activities.

## Results

### Mitochondrial DNA sequence variation

A total of 823 bp of the mitochondrial DNA control region was analyzed after discarding a 82 bp region at the 5′ extreme with variable number of repeats within (heteroplasmy) and across individuals. Since the homology between repeats could only be unequivocally established for the flanking region and the last repeat, the rest of repeats were discarded. Analysis of the 42 parental stock specimens revealed 14 variable sites (1 transversion, 13 transitions), which defined 6 haplotypes (Table 1). No changes in haplotype and nucleotide diversities were found when comparing the 2005 sample with that of the individuals presently alive (Table 2). However, all F1 samples showed a 50% decrease in haplotype diversity (from 6 to 3 haplotypes at each sample), which was more apparent at the Orzinuovi F1 sample (H_d_ =  0.153). A two-fold drop in nucleotide diversity was also observed in all F1 samples (Table 2).

**Table 1:** List of haplotypes identified on the basis of 823 bp of the mitochondrial DNA control region in *A. naccarii*.

Haplotype	44	78	126	160	163	168	171	220	248	280	364	461	545	546	Wild	Wild- Present	F1-Treviso	F1-Piacenza	F1- Orzinuovi
**Haplotype 1 (Po1)**	**G**	**T**	**G**	**T**	**C**	**C**	**T**	**C**	**A**	**G**	**C**	**T**	**T**	**C**	2	2	-	-	-
**Haplotype 2 (Po1)**	A	*	A	*	*	*	C	*	*	*	*	*	*	*	10	4	41	35	46
**Haplotype 3 (Po1)**	A	*	A	*	*	T	C	*	*	*	*	*	*	*	13	9	7	4	3
**Haplotype 4 (Po1)**	A	*	A	*	T	*	*	*	*	A	*	*	*	*	1	1	-	8	-
**Haplotype 5 (Po2)**	A	C	A	C	*	*	*	T	G	*	T	C	C	G	12	6	2	-	-
**Haplotype 6 (Po2)**	A	C	A	C	*	*	*	T	*	*	T	C	C	G	4	2	-	-	1
															(42)	(24)	(50)	(47)	(50)

Vertical numbers indicate variable positions for haplotype 1. Haplogroups Po1 and Po2 are indicated in parenthesis. The number of individuals showing each haplotype is reported for all samples.

**Table 2:** Diversity indices for all samples including number of individuals (N), number of segregating sites (S), number of singletons (S_I_), number of haplotypes (H), haplotype diversity (H_d_), nucleotide diversity estimated from number of segregating sites (θ_w_), and nucleotide diversity estimated from mean number of pairwise differences (θ_π_).

Sample	N	S	S_I_	H	H_d_	θ_w_	θ_π_
**Wild**	42	14	2	6	0.772	0.004	0.006
**Wild-Present**	24	14	2	6	0.786	0.005	0.006
**F1-Treviso**	50	10	0	3	0.313	0.002	0.001
**F1-Piacenza**	47	4	0	3	0.418	0.001	0.001
**F1-Orzinuovi**	50	9	8	3	0.153	0.002	0.001

While haplotype 2 represented 23.8% of the parental F0 haplotypes, this haplotype was found in similar high frequencies in the F1 stocks (Treviso: 82%; Piacenza: 74.5%; Orzinuovi: 92%). Accordingly, pairwise genetic differences were higher between F0-F1 samples (F_ST_ =  0.266–0.321) than among F1 samples (F_ST_ =  0.003–0.087) (Table 3).

**Table 3:** Matrix of pairwise F_ST_ values among samples at mitochondrial DNA (below diagonal) and at 8 microsatellite loci (above diagonal).

Sample	Wild	F1-Treviso	F1-Piacenza	F1-Orizinuovi
**Wild**	-	0.237*	0.262*	0.207*
**F1- Treviso**	0.266*	-	0.203*	0.033*
**F1- Piacenza**	0.293*	0.067*	-	0.090*
**F1- Orzinuovi**	0.321*	0.003	0.087*	-

At the Piacenza F1 stock, 3 out of the 50 individuals analyzed presented an allochthonous haplotype, not present in the parental stock, and highly divergent from any *A. naccarii* control region sequence. A Blast search revealed the three haplotype sequences as belonging to the white sturgeon *Acipenser transmontanus*, a North American species frequently employed in Italy for aquaculture production of caviar. Consequently, these three individuals were excluded from further analyses.

Phylogenetic network analyses confirmed the presence of two clearly distinct haplogroups, designated as Po1 and Po2 in the previous study of Ludwig *et al*. [12], in which a sub-set of the same parental stock was analyzed (data not shown). A 1.1% sequence divergence was found between haplogroups.

### Microsatellite variation

**Figure 1:**
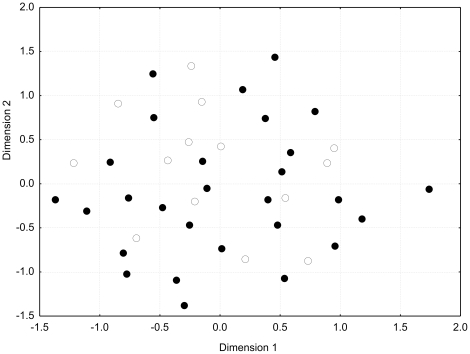
Plots of the values of the first and second principal coordinates obtained from pairwise genetic distances at 24 microsatellite loci among all individuals from the parental stock (N = 42) plotted according to mtDNA haplogroup, Po1 (black circles) and Po2 (white circles). Stress value =  0.29.

The 24 microsatellite loci analyzed using a band sharing approach were moderately polymorphic, revealing 2 to 16 alleles per locus among the 42 individuals from the original parental stock (Table 4). Each individual was characterized by a distinct multilocus profile when combining data of all 24 loci. We conducted a Multidimensional Scaling analysis using genetic distances based on band sharing values at 24 loci among all individuals from the parental stock (Figure 1). From the figure, it seems clear that no separation exists between individuals corresponding to the two Po mtDNA haplotypes (Po1 and Po2).

**Table 4:** List of microsatellite loci analyzed in *A. naccarii* including repeat motif, size range (in bp), number of alleles per locus (NA) and maximum number of alleles per locus (MNA), and species in which the microsatellites were originally developed.

Locus	Motif	Size	NA/MNA	Species	Reference
**An1**	(AATC)_6_	180–210	3/3	*A. naccarii*	Zane et al. 2002
**An16**	(ATCT)_24_	171–217	16/4	*A. naccarii*	Zane et al. 2002
**An20**	(ATCT)_10_(TG)_5_	159–213	10/4	*A. naccarii*	Zane et al. 2002
**AnacB11**	(CA)_9_AA(CA)_10_	132–162	8/4	*A. naccarii*	Forlani et al. 2007
**AnacA6**	(CA)_15_	289–313	9/4	*A. naccarii*	Forlani et al. 2007
**AnacB3**	(CA)_14_	359–361	5/3	*A. naccarii*	Forlani et al. 2007
**AnacB10**	(GTT)_17_	212–258	11/4	*A. naccarii*	Forlani et al. 2007
**AnacB7**	(GA)_23_	152–176	11/4	*A. naccarii*	Forlani et al. 2007
**AnacC11**	(TCTA)_12_	167–193	9/4	*A. naccarii*	Forlani et al. 2007
**AnacG8**	(CA)_13_	130–140	3/3	*A. naccarii*	Forlani et al. 2007
**AnacE4**	(CA)_20_	326–354	10/4	*A. naccarii*	Forlani et al. 2007
**AnacA1**	(AC)_9_	143–145	2/2	*A. naccarii*	Forlani et al. 2007
**AnacD3**	(TCTA)_21_	135–203	14/7	*A. naccarii*	Forlani et al. 2007
**LS-19**	(TTG)_9_	126–138	3/3	*A. fulvescens*	May at al. 1997
**LS-34**	(GTT)_10_	141–150	3/3	*A. fulvescens*	May at al. 1997
**LS-39**	(GTT)_10_	116–154	8/3	*A. fulvescens*	May at al. 1997
**AfuG56**	(AAAC)_9_	263–279	5/2	*A. fulvescens*	Welsh et al. 2003
**AfuG112**	(GATA)_12_GACA(GATA)_6_	222–253	8/2	*A. fulvescens*	Welsh et al. 2003
**AfuG63**	(AAAC)_8_	118–144	4/2	*A. fulvescens*	Welsh et al. 2003
**Spl120**	(TATC)_15_	263–303	9/3	*S. platorynchus*	McQuown et al. 2000
**AoxD241**	(TAGA)_36_	156–198	11/4	*A. oxyrinchus*	Henderson-Arzapalo & King 2002
**AoxD64**	(TAGA)_16_	216–252	8/4	*A. oxyrinchus*	Henderson-Arzapalo & King 2002
**AoxD161**	(CTAT)_15_	111–155	9/4	*A. oxyrinchus*	Henderson-Arzapalo & King 2002
**AoxD234**	(TAGA)_17_	215–275	14/4	*A. oxyrinchus*	Henderson-Arzapalo & King 2002

At 8 microsatellite loci used for characterization of the F1 groups, mean number of alleles across the F1 stocks (Treviso: 9.63; Piacenza: 8.88; Orzinuovi: 10.00) was lower in comparison with the F0 stock (11.13) (Table 5). Accordingly, the F0 stock showed a lower average band sharing (40%) in comparison with the F1 stocks (Treviso: 54%; Piacenza: 59%; Orzinuovi: 51%). Differences in allele distribution were significantly different between F0 and F1 stocks (F_ST_ =  0.207–0.262; p<0.001) and among F1 stocks (F_ST_ =  0.033–0.203; p<0.001) (Table 3).

**Table 5:** Total and mean number of alleles and average band sharing (BS) values at 8 microsatellite loci for all samples.

Locus	Wild (N = 42)	Wild-Present (N = 24)	F1-Treviso (N = 50)	F1-Piacenza (N = 47)	F1-Orzinuovi (N = 50)
**An16**	16	12	12	10	15
**An20**	10	9	9	8	8
**AnacB10**	11	10	9	9	9
**AnacD3**	14	13	11	12	13
**AnacE4**	10	8	10	8	8
**AfuG56**	5	4	4	4	4
**Spl120**	9	9	9	8	9
**AoxD234**	14	14	13	12	14
**Mean**	**11.13**	**9.88**	**9.63**	**8.88**	**10.00**
**BS**	**0.40**	**0.34**	**0.54**	**0.59**	**0.51**

**Figure 2:**
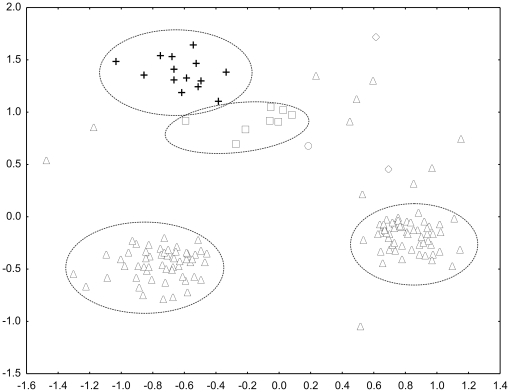
Plots of the values of the first and second principal coordinates obtained from pairwise genetic distances at 8 microsatellite loci among all F1 stocks (Treviso, Piacenza, Orzinuovi) plotted according to mtDNA haplotype: haplotype 2 (triangles), halotype 3 (crosses), haplotype 4 (squares), haplotype 5 (diamonds), haplotype 6 (circles). Stress value =  0.14.

We conducted a Multidimensional Scaling analysis using genetic distances at 8 microsatellite loci among all F1 stocks (Figure 2). When plotting the values of the first and second principal components, individuals clustered according to mtDNA haplotype. The MDS analysis showed two clear distinct groupings for those individuals corresponding to haplotypes 3 and 4, respectively, plus two groupings of highly related haplotype 2 individuals, which suggests that those groups are constituted by sibs (brothers and sisters).

### Parental allocation

Table 6 summarizes results from parental assignment of all the F1 progeny (N = 147) obtained by comparison of microsatellite profile between progeny and all possible parent-pairs. Mitochondrial data were also used to solve ambiguities due to multiple parental allocations, thus compatible parent-pair profiles were discarded if there was no concordance between maternal and F1 mitochondrial haplotype. 134 out of 147 F1 individuals were unequivocally assigned to a single parent pair, which corresponds to an allocation success of 91.2%. No cases of multiple parent assignment were observed. The remaining 13 individuals presented a profile that was incompatible with all possible parent-pairs.

**Table 6:** Parental allocation of all F1 stocks (Treviso, Piacenza, Orzinuovi) to the Azienda Agricola VIP F0 parental stock.

F1	Allocation	
	Certain	No allocation
PC01 PC02 PC03 PC05 PC06 PC14 PC16 PC17 PC18 PC19 PC20 PC22 PC23 PC24 PC26 PC27 PC30 PC31 PC33 PC34 PC36 PC37 PC38 PC39 PC40 PC45 PC46 PC48 PC50 TV03 TV04 TV08 TV13 TV18 TV21 TV25 TV28 OR134 OR136 OR142 OR153 OR157 OR162 OR163 OR165 OR169 OR170 OR171 OR177 OR178 OR196 OR198(N = 52)	Matto(♂) X Naccs8(♀)	
PC04 PC08 PC13 PC15 PC29 PC35 PC47(N = 7)	Naccs13(♂) X Naccs15(♀)	
PC07 PC011 PC43 PC44 TV02 TV05 TV06TV10 TV34 TV36 TV47 OR188 OR190 OR203(N = 14)	NaccS6(♂) X Naccs7(♀)	
PC21 PC25 PC42 TV07TV11 TV12 TV14 TV15 TV16 TV17 TV19 TV20 TV22 TV23 TV24 TV26 TV27 TV29 TV30 TV31 TV32 TV33 TV35 TV37 TV38 TV39 TV40 TV41 TV42 TV43 TV45 TV46 TV48 TV49 TV50 OR1 OR5 OR14 OR17 OR18 OR22 OR37 OR38 OR41 OR45 OR56 OR85 OR91 OR92 OR114 OR115 OR118 OR122 OR129 OR130 (N = 55)	NaccS18(♂) X Pelvienne(♀)	
OR149 OR150 OR173 (N = 3)	Naccs31(♂) X NaccS8(♀)	
TV44 (N = 1)	Matto(♂) X O2(♀)	
TV01 (N = 1)	Matto(♂) X Pelvienne(♀)	
OR181 (N = 1)	Naccs13(♂) X Ditata(♀)	
OR148 (N = 1)	Matto(♂) X Raspo(♀)	
PC09 PC28 PC32 PC41 TV09 OR175OR176 OR182 OR208 OR210 OR212 OR214(N = 12)		No compatible parent-pairs

Parental allocation data indicated the F1 stocks to result from few founders, only five males and six females, in a total of nine pairings. As shown in Table 6, 131 out of the 147 F1 individuals were allocated to five single families, notably to two families with a progeny of 55 and 52 individuals, respectively. The remaining four individuals were single representatives of their respective families.

### Choice of candidate breeders

**Figure 3:**
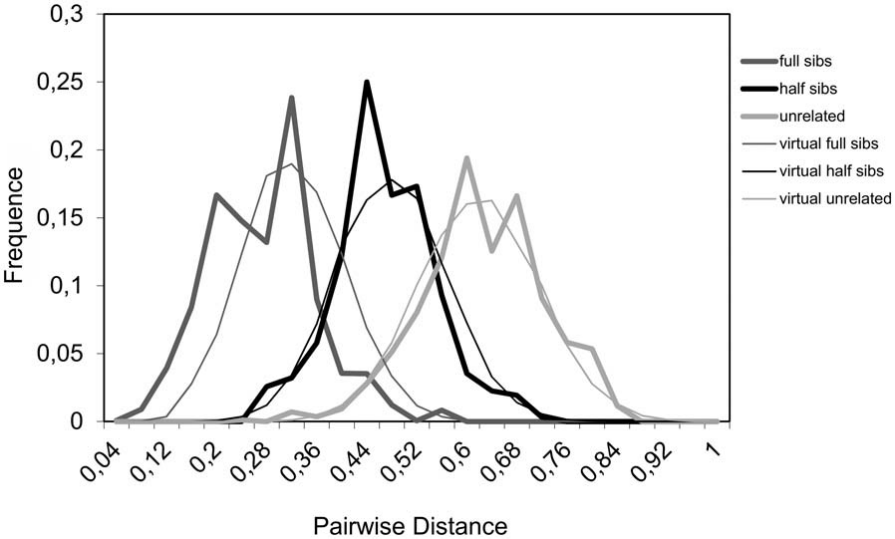
Observed and simulated observed distributions of pairwise genetic distances in the different groups of relatives (full sibs, half sibs, unrelated).

Figure 3 shows the distribution of pairwise genetic distances observed between F1 full sibs, half sibs as well as unrelated individuals following parental assignment. As expected, genetic distances were lower between full sibs than between unrelated individuals, while half sibs showed intermediate values. Little overlapping was found among the different groups of relatives, which allowed us to define a threshold value above which the chance of excluding a related individual is >99%. The threshold value was 0.50 for full sibs and 0.70 for half sibs.

A high correspondence was observed between observed and simulated distributions of pairwise genetic distance (Figure 3). Using the simulated data, the threshold values were 0.50 and 0.67 for full sibs and half sibs, respectively, which coincide with the observed ones.

## Discussion

### Genetic variability

The parental wild stock showed a moderately high level of genetic variation as indicated by high mtDNA haplotype diversities and the observation of a distinct microsatellite profile for each F0 individual. Since the bottleneck is recent, it is possible that the loss of genetic diversity is not yet detectable. This observation corroborates other recent empirical studies suggesting that long generation times may act as a buffering effect contributing to a reduction in the pace of genetic diversity erosion in theatened species [14]–[16]. Namely, a long lifespan of approximately 40–50 years and age at maturity of about 7–14 years in Adriatic sturgeon (Giovannini, pers. comm.) result in a long generation time that could slow down or moderate the rate of loss of genetic diversity.

Comparison of the 2005 and the present parental stock showed that a considerable part of genetic variability has been retained, despite the recent drop in population numbers (from 42 to 24 individuals). No loss of haplotypes was observed at the mtDNA level, with similar haplotype and nucleotide diversity values found between samples, while microsatellite data only showed a slight reduction in number of alleles in the present sample.

By contrast, a drastic decline in genetic variability based on the mtDNA was found between F0 and F1 groups, which was less apparent at the microsatellite level. Both haplotype and nucleotide diversity diminished by 50%, from 6 haplotypes observed in the parental stock to 3 haplotypes in each F1 stock. The loss of genetic variation is probably a consequence of relatedness, with F1 individuals being more related than the parental wild stock. Parentage allocation assigned all F1 progeny to only five males and six females (nine pairings), and about 95% of the allocated individuals were assigned to four single families, with two of the crosses producing 55 and 52 of the F1 individuals, respectively.

Mitochondrial DNA analysis also detected the presence of three individuals with a highly divergent haplotype, identified as belonging to the North American species *A. transmontanus*, frequently used in Italy for aquaculture caviar production. This exotic species is reared at the Azienda Agricola VIP plant where *A. transmontanus* x *A. naccarii* hybrids are produced. We hypothesize that three of those hybrid individuals have been accidentally introduced in the rearing tanks with the F1 *A. naccarii* stocks. Those hybrids have been pointed out to the operators in the facilities and appropriately removed from the stock.

In agreement with the study of Ludwig *et al.* [12] conducted on a sub-set of the F0 parental stock, two highly differentiated haplogroups (Po1 and Po2) were detected on the basis of mtDNA. Samples from the same haplogroup did not cluster together in a Multidimensional Scaling analysis, which confirms the results from Ludwig *et al.* [12] where nuclear data only partially validated the existence of the two groups. The discordance between microsatellites and control region might be explained by the homogenizing effect of recombination at nuclear level, which does not occur in mitochondrial genomes.

### Parental allocation success

The combination of eight highly polymorphic nuclear loci with additional mtDNA information resulted to be highly efficient in order to assign the F1 progeny to the correct F0 parental pair. All positive allocations were unequivocal, with no ambiguities due to multiple allocations. Only 13 out of 147 F1 individuals presented a profile that was incompatible with all possible parent-pair combinations, which suggests that those parents died between 1988 and 2005 and were not included in our sampling. It should be noted that the high allocation power in our analysis might be due to the limited number of parent pairs, and that the assignment of a progeny to a larger parental stock might require a higher number of marker loci.

Besides its applicability in the selection of candidate breeders for artificial reproduction, parental assignment can be useful for the identification of individuals recaptured in the wild. A positive match would indicate an F1 produced at the Orzinuovi plant and released into the river Po as part of the frequent restocking activities conducted in the region since the late 1980s. A negative match would suggest either an individual of wild origin not derived from the Orzinuovi stock or an F1 produced at Orzinuovi whose parents died in the period 1988–2005 and were not included in our sampling.

### Management implications

A first mandatory step toward an adequate restoration program for the Adriatic sturgeon is to conduct a complete and thorough genetic tagging of all the individuals currently reared in captivity. This includes (i) the three F1 broodstocks of Treviso, Piacenza and Orzinuovi, of which a sub-set of 150 individuals has been genetically analyzed in the present study; (ii) additional F1 individuals produced by the Azienda Agricola VIP through the years that have been maintained in the facilities.

As our study pointed out, the F1 stocks have only retained a part of the genetic variation present in the original F0 stock due to the few founders used for reproduction, which underlines how little attention was paid to genetic diversity at the time of the establishment of those F1 stocks in the late 1980s/early 1990s. In that respect, a second action urgently needed is the improvement of the current F1 stocks by incorporating new founders with the aim of maximizing the amount of genetic diversity transmitted to coming generations.

Because of its higher genetic variability, the F0 parental stock should have priority over the F1s in any breeding scheme. A very low effective population size (<20) was recently estimated for the founder population of the Atlantic sturgeon (*A. oxyrinchus*) in the Baltic rivers [17]. For this reason the availability of 25 Adriatic sturgeon of wild origin can potentially provide enough variability if correctly managed. Those F0 animals might now probably be around 35 years of age, given that they were taken as juveniles from the wild and transferred to the fish plant in the late 1970s. No data are available on the longevity of the species but it is possible that the mortality observed in recent years at the fish plant might be due to senescence. A realistic scenario for the management of the breeding stock should prioritize the use of F0 individuals as founders while they are still alive, but at the same time, it should contemplate that the F0 generation might be gone in a matter of years, and plan out the incorporation of the F1 into the breeding program, first as F0×F1 crosses and eventually as F1×F1 crosses in due time.

An effective strategy to increase genetic variability would be to identify those F0 individuals that have not yet contributed to the F1 progeny and prioritize their use as founders. It would also be effective to rationalize the genetic composition of the F1 broodstocks by reducing the number of sibs and replacing some sibs by individuals from under-represented families.

**Figure 4:**
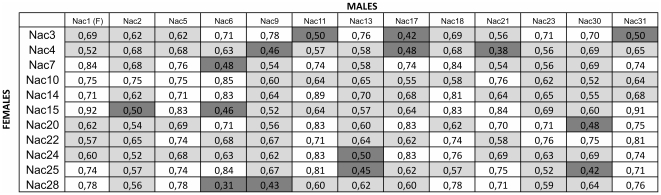
Example of pairwise genetic distance matrix to be used for choice of candidate breeders. Colour-code refers to risk of inbreeding: white (low/unrelated individuals), light grey (medium/half sibs), dark grey (high/full sibs).

The genetic characterization of all the breeders will allow to set up an optimal long-term breeding program aiming at maximizing genetic diversity and minimizing inbreeding with the ultimate goal of re-introducing the Adriatic sturgeon in its natural habitat. We propose a standardized and user-friendly tool that can be easily employed by operators in aquaculture facilities when setting up captive breeding programs. Following genotyping of all individuals using microsatellite markers, a matrix is constructed summarizing genetic distances between all possible individual pairs based on band sharing values. Using the previously-identified threshold values (<0.5 for full sibs, between 0.5 and 0.7 for half sibs, >0.7 for unrelated individuals), the relationship among all possible parent-pairs and their adequacy as future breeders is colour-coded according to inbreeding risk: white for unrelated individuals with low risk of inbreeding, light grey for half-sibs with medium risk, and, dark grey for full-sibs with high inbreeding risk. Figure 4 shows an example matrix including all possible crosses among the individuals of the parental stock. The same approach was used to construct an analogous matrix considering also the F1 putative breeders.

The implementation of this intuitive and easy-to-use method can facilitate the choice of candidate breeders that secure the use of non-related parents in captive breeding programs. For example, assuming that all breeders are tagged with a microchip, given an ovulating female, operators simply need to cross-check in the distance matrix which of the candidate males are adequate or not according to the colour-code, and a matching male can easily be selected for milt collection.

The method is specifically designed for polyploid species and the band sharing approach is used due to the impossibility to infer the true genotype, thus it can be applied to all polyploid sturgeon species. The set up of a simple standardized protocol might be particularly relevant in regions with scarcity of facilities or lack of adequate expertise for the management of genetic diversity in sturgeon hatcheries. This is the case of the Caspian Sea region, where many sturgeon species with high commercial value for caviar production occur. The technical capacity within Caspian Sea caviar exporting countries to perform genetic research is variable. For instance, conservation activities in the Russian sturgeon *A. gueldenstaedtii* have been conducted through artificial reproduction and restocking practices in Russia and Iran, while other countries in the region (Azerbaijan, Turkmenistan, Kazakhstan) are only at the starting point in the development of conservation plans. Those countries do not currently have the capacity to conduct genetic analysis but plan to set up genetic laboratories shortly, and would greatly benefit from a simple and effective protocol for the genetic characterization of polyploid sturgeons like the one presented in this paper.

Due to the high rate of mortality observed in the parental stock, decimated from 42 individuals in 2005 to 24 individuals at present, the establishment of an adequate *ex situ* restoration program utilizing genetic information and aiming at maximizing the original genetic diversity is extremely urgent. There is no evidence of natural reproduction, which makes the Adriatic sturgeon effectively dependent on captive breeding programs. In parallel with all genetic work, a successful reintroduction of Adriatic sturgeons obtained and raised in captivity into the wild can only be achieved if factors affecting the natural habitat are monitored, including restoration of spawning sites an establishment of fish passages at dams. Detailed guidelines and recommendations are listed in the Ramsar Declaration on Global Sturgeon Conservation [6]. This constitutes the last chance for this endemic and critically endangered species.

## Materials and Methods

### Sampling collection

The University of Padova ethic board C.E.A.S.A. (Comitato Etico di Ateneo per la Sperimentazione Animale) exempted this study from review because it was an extra moenia activity. Nevertheless we tried to minimize the impact of sample collection on the animals. All samples were collected in aquaculture facilities by the owners. Fish were kept into the pond water to minimize stress and were released immediately after sample collection, which consisted of painless clippings from the caudal fin. No mortality, pain or stress was observed.

Samples of Adriatic sturgeon (*A. naccarii*) were obtained at Azienda Agricola VIP (Orzinuovi, Brescia) from the parental stock constituted by individuals caught in the river Po and transferred to the fish plant in 1977, which is the last remaining Italian population of wild origin.

A total of 42 parental F0 individuals have been analyzed. In 2005, all surviving animals from the original parental stock at that time (33 individuals, 15 males and 18 females) were sampled for genetic analysis and tagged for the first time with a microchip. The sample set was completed with additional individuals from a previous sampling conducted in 1999/2000, in which a sub-set of the parental stock was sampled following individual identification ‘by eye’. Nine of the individuals sampled in 1999/2000 were not present in the 2005 sampling as they died sometime in the period 2000–2005. Those animals were added to the 2005 set as they might have been used as founders in producing the F1 stocks. Following a period of high mortalities in recent years, the current stock has been decimated to 24 individuals (13 males and 11 females). All analyses have been conducted separately for the full data set (referred as Wild; N = 42) and considering only those individuals presently alive (referred as Wild-Present; N =  24).

Additionally, genetic analysis included a total of 150 individuals corresponding to the three F1 stocks obtained from unrecorded artificial reproduction using the Azienda Agricola VIP parental stock of wild origin, currently reared as future breeders at three aquaculture plants in Treviso, Piacenza and Orzinuovi (N =  50 at each site).

Finally, two complete families each composed by both parents plus 10 fingerlings were also sampled following artificial reproduction, and genotyped at genetic markers in order to test the reliability of the parental allocation procedure.

### Mitochondrial DNA analysis

Genomic DNA was extracted from fin clips (10–100 mg) using the DNAeasy Blood and Tissue Extraction kit (Qiagen). The entire mitochondrial control region including partial sequences of the flanking tRNA genes was amplified and sequenced for all F0 and F1 individuals. Primers used matched the proline (Pro1F: 5′-CACCCTTAACTCCCAAAGC-3′) and phenylalanine tRNA (Phe1R: 5′- CCCATCTTAACATCTTCAGT-3′), respectively. PCR reactions consisted of about 10 ng template DNA, 2.5 µl 10x buffer, 200 µM of each dNTP, 1.5 mM MgCl_2_, 10 pmol of each primer, 1 U Taq DNA polymerase (GE Healthcare Biosciences), and water up to 25 µl. PCR conditions were as follows: 3 min at 94°C, 35 cycles of 30 sec at 94°C, 45 sec at 53°C and 1 min at 72°C, and final elongation for 5 min at 72°C. Following enzymatic purification with ExoSAP-IT™ (Usb), sequence reactions were performed using primer Phe1R, which allowed to obtain unambiguous sequences of the entire control region. By contrast, the use of primer Pro1F yielded sequencing failures due to the co-sequencing of different heteroplasmic variants linked to a 82 bp tandem repeat region located at the 5′ region [18]. Sequencing reactions were performed using an ABI Prism3730XL automatic sequencer.

### Microsatellite analysis

All F0 individuals were analyzed at a total of 24 polymorphic microsatellite loci (Table 4): 13 isolated *ex novo* in Adriatic sturgeon [19]–[20], and 11 originally isolated in other sturgeon species, *A. fulvescens* [21]–[22], *Scaphirhynchus platorynchus* [23] and *A. oxyrinchus* [24].

All F1 individuals were analyzed at a sub-set of 8 microsatellite loci (AN16, An20, AnacB10, AnacD3, AnacE4, AfuG56, Spl120, AoxD234). In order to achieve maximum scoring accuracy, analysis was conducted in parallel in two separate laboratories, including independent tissue extractions for each animal. Microsatellite loci were amplified following the conditions reported in the original references (Table 4).

### Data analysis

Mitochondrial DNA sequences were aligned using CLUSTAL W [25]. For all F0 and F1 groups, genetic diversity was measured using haplotype and nucleotide diversity estimated from number of segregating sites and mean number of pairwise differences in DnaSP v. 5 [26]. Pairwise F_ST_ values were calculated in ARLEQUIN [27]. A phylogenetic network of mitochondrial DNA haplotypes was constructed using the statistical parsimony approach of Templeton *et al*. [28] using TCS v. 1.13 [29].

Microsatellite data were analyzed using a phenetic approach, with microsatellite alleles scored as dominant markers. Interpretation of microsatellite patterns in tetraploid species is not straightforward due to the presence of multiple alleles at each locus that can be present in more than one copy, thus the true genotype cannot be accurately resolved. Alternatively, some authors propose to infer allele frequencies from gene dosages, assuming a correspondence between number of allele copies and peak heights in the chromatogram [30]. No such correlation was observed in the case of *A. naccarii*, e.g. the four peaks for a complete heterozygous genotype did not always show the same height even if they correspond to a single copy of each allele, thus it was not possible to estimate gene dosages on the basis of peak heights. Using the classic bandsharing approach, microsatellite data were considered as presence/absence of bands, disregarding the number of alleles present in each individuals. Data from all microsatellite loci were combined by creating individual profiles in which bands were coded as a string of 0s (absent) and 1s (present). Band sharing (BS) between individuals was used as a measure of similarity. Genetic distances were calculated as 1-BS, and graphically represented by Multi-Dimensional Scaling (MDS) analysis using STATISTICA version 7.1 (StatSoft). Pairwise F_ST_ values were calculated in ARLEQUIN [27].

### Allocation procedures

Assignment of F1 individuals to the correct F0 parental pair was conducted in two steps. First, the microsatellite profiles of all putative parent-pairs were combined and compared with the profile of each F1 individual to be allocated. Parent-pairs not showing all bands observed in the F1 were discarded as possible parents. Among all compatible pairs, a second selection step consisted in comparing the mitochondrial DNA haplotype between mother and progeny. Prior to the application of the allocation procedure, accuracy was tested on 20 F1 individuals (two groups of full-sibs) of known pedigree. Using the 42 individuals of the F0 group as putative founders, all 20 F1 individuals were allocated to the correct parent pair with a 100% accuracy.

### Choice of candidate breeders

Genetic distance (1-BS) was calculated between all pairs of full sibs, half sibs and unrelated individuals. Using the observed distribution of genetic distances within groups, a threshold value was established above which the probability to exclude that two individuals are related (full or half sibs) is >99%.

Simulations were used to verify if the observed distributions of pairwise genetic distances in the different groups of relatives (full sibs, half sibs, unrelated) correspond to the expected ones. Diploid gametes drawn from all F0 animals were combined to produce a simulated tetraploid genotype for 8 independent microsatellite loci for each F1 individual. In order to take into account the tetraploid nature of the species, two alleles per parent were randomly drawn. When less than four alleles were observed at a given locus, the possibility to segregate the same allele twice was accepted. With this approach we assumed a tetrasomic pattern of inheritance, in which the different homolog chromosomes can be transmitted to gametes in all possible combinations. The obtained microsatellite profiles were used to reconstruct the distribution of genetic distances (1-BS) in simulated full sibs, simulated half sibs and simulated unrelated individuals. For each group, the threshold value that excluded related individuals with a >99% chance was calculated using the simulated data, and compared with the threshold values previously obtained with the observed data. The program used for virtual offspring simulation and estimation of pairwise distances within different classes of relatedness is available upon request, as well as the one used for parental allocation.
